# A Summer Holiday in North-West America

**Published:** 1909-08-07

**Authors:** 


### The Practitioner's Relaxations.

### A SUMMER HOLIDAY IN NORTH-WEST AMERICA.

Calgary was in the clutches of a venomous blizzard when the west-bound train clanged its monotonous bell and steamed out to the accompaniment of the flagman's cry "All aboard, right now." We headed for the mountains, and long before we reached them the jagged line of white-capped peaks seemed to tower above and around us. So gradual was the ascent that we were surprised when we really found ourselves in the midst of their ravines, great rocky masses rising up on every side. The patient train wound in and out amongst buttress and boulder and precipice, along a narrow trail through the Kicking Horse Pass that is a triumph of engineering skill and bravery. We were still rising into the heights when darkness fell.

Sunrise and morning found us well in British Columbia: still mountains, still rocks and ravines; but now we mostly followed the shores of great silent lakes, with here and there the orchard of a lonely fruit grower perched between rocky hillside and water's edge. Brilliant orange and scarlet and yellow coloured forest and mountain, but desolate and unpopulated. Now and again we passed a lumber camp, where Hindoo and Japanese work side by side with the white man; sometimes a little village with orchards and fields, but mostly Nature was alone. For many miles we wound along the banks of the great Fraser River, and often on the hillside a solitary grave told of a prospector's end or a trapper's fatality. Towards evening, suddenly, from out of the wilderness of fir-clad Selkirks we whirled down on our terminus, Vancouver, a charming city of trees, mountains, waters, and mist. The shops, streets, and bustle spoke of civilisation again, and came refreshingly after many months of a western village. Stone buildings and paved streets looked solid, comfortable, and home-like after the shabby wooden shacks and stores of the Prairie Provinces; and well-kept gardens and parks told of an English love of order and decency. Stanley Park, one of the beauties of the city, is a piece of the wild British Columbian land preserved and trained in the midst of shop and street. Enormous trees, maples, sycamores, and oaks reach down to the waters of the Sound, which nearly encircles the Park; streams, waterfalls, dells, woodland paths, and luxuriant undergrowth make scenes of surprising beauty. Many varieties of birds and waterfowl, also buffalo and elk, are to be seen, and the ferns would be a botanist's delight.

It was almost with regret that I had to continue my journey by steamer across Puget Sound to Seattle. There we found one of the most enterprising cities in the world; wherein, however, the citizens are still dissatisfied, for the site is very hilly, and only allows of street cars on the cable system. They have therefore engaged in the Herculean task of levelling it to suit their wishes. Enormous banks are washed away by water pumped from the Sound, and the hollows filled up with the superfluous earth; mountains indeed being levelled, and the valleys made plain. This wonderful labour is taking place all over the city at enormous cost, but it pleases our cousins to work their will in Nature as in commerce. The winding bays, high mountains, and extensive forests that surround Seattle were mostly enveloped in mist and fog, but now and again we got glimpses of such scenery as would make any city famous. We obtained a better idea of the beauty of Washington State as the steamer threaded its way down Puget Sound to the Pacific, with Vancouver Island on the north.

During our southward course we were long in sight of land, and lazily watched the purple mountains rising out of the blue foam-flecked ocean, with here and there green and indigo patches where forests clothed the hillsides, and over all a cloudless azure sky. Whales we saw on two occasions, spouting fountains of water, and frequently gambolling schools of porpoises passed the steamer within a few yards. Early on the fourth morning we woke to find ourselves at anchor, and watched the sun rise through mist and fog-bank on an almost foreign-looking town; buildings of many colours crowded up and down the steep hills that reached to the water's edge; masses of tall, dark trees threw deep shadows on street and house; and surrounding all was a yellow, sandy, desert-like land. We soon found that San Francisco is nevertheless very modern, very American, very up-to-date. It has largely been rebuilt since the earthquake and fire of 1906, and but few signs remain of that terrible devastation. The streets are for the most part very crowded and busy, but here and there one comes unexpectedly upon quaint out-of-the-world corners; the peaceful old church of the Franciscan missionary fathers; the quiet cemetery with palms and cypresses and rose-trees; an occasional low Spanish house left by chance among the brick and stone of modern days.

There are many attractions in San Francisco, but naturally our first excursion was across the harbour, past its islands and fortifications, to Berkeley, where the Universtiy of California is situated. Passing through an extensive park of ancient trees, oaks, sycamores, and many others which were unfamiliar to me, we finally reached the well-kept grounds surrounding the numerous scattered buildings of the University; long, low battlemented buildings of dark brick, all almost covered with Virginia and other creepers, now scarlet and orange with their autumn leaves. Each department appeared to be quite distinct, with well-equipped laboratories and necessary ante-rooms. Owing to the hurried nature of our visit, we were unable to see over the University as we should have wished.

Another of San Francisco's beauties is the Golden Gate Park, the third largest artificial park in the world. It was wonderful to look on those massive trees, luxuriant vegetation, flowers and shrubs, and to realise that only a few years ago it had been a desert of shifting sand dunes. The Golden Gate itself is the narrow opening of the harbour with its forts on either side, and as the steamer passed through we met the heavy rollers of the Pacific. For the rest of our journey we were in view of the Californian coast, great rocky hills rising precipitously from the sea, for the most part bare of vegetation. Here and there were sheltered bays with sandy beaches, and small towns clustering amid palm and eucalyptus and pepper trees on their shores. At some of these we put in for a few hours; Santa Barbara, with the Santa Inez mountains for a background, and one of the chain of old mission churches on the hillside; Redondo, with beautiful gardens where humming-birds, bees and butterflies disport themselves all winter; San Pedro, a busy little town with its eye on the present opportunity, and holding no links with past or future; and finally, rounding Point Loma, with the golden dome of the Theosophical College glittering in the setting sun, we entered the long, winding harbour of San Diego. Here, a partially Latin town, the southern love of art and colour shows in many ways; picturesque buildings, new ones built in the old Spanish architecture, old ones rebuilt and restored; a plaza, with well-kept grass and huge palm-trees, standing in one of the busiest thoroughfares; gardens with roses and passion-flowers, bushes of purple bougainvillæa and hedges of geranium and fuchsia. Continuous sunshine, brilliant blue skies, and warm moist breezes from the ocean are the lot of this fortunate town in which I ended my journey. E. M. M.

